# Is cannabis a slippery slope? Associations between psychological dysfunctioning, other substance use, and impaired driving, in a sample of active cannabis users

**DOI:** 10.1371/journal.pone.0310958

**Published:** 2024-10-09

**Authors:** Steven Love, Bevan Rowland, Kerry Armstrong

**Affiliations:** MAIC/UniSC Road Safety Research Collaboration, University of the Sunshine Coast, Sippy Downs, Queensland, Australia; NYU Grossman School of Medicine: New York University School of Medicine, UNITED STATES OF AMERICA

## Abstract

Cannabis is a gateway drug that can lead to the engagement of other substances. Psychological dysfunctioning and dependence have been highlighted as primary components to substance misuse. The purpose of this study was to investigate what aspects of cannabis use and psychological dysfunctioning are associated with the engagement of other substances and impaired driving. Subject to screening, 200 active adult cannabis users completed an online survey. Existing data involving non-cannabis users (*N* = 833) were also implemented as comparative data. The comparisons suggested that cannabis users were far more likely to have used other drugs in the past 12 months, compared to non-cannabis users. Bivariate correlations and multiple regressions indicated that the degree of cannabis use and likely dependence, psycho-social motives for using cannabis, emotion dysregulation, and psychopathology were positively associated with the frequency of using and driving on other substances. Finally, an ANOVA demonstrated that outside of age, there were no apparent differences in substance use behaviours, motives for using cannabis, and psychological dysfunction, between medicinal and black-market cannabis users. These findings highlight the potential benefits of incorporating self-regulatory concepts into current road safety initiatives, which aim to reduce the interconnected issue of substance misuse and impaired driving behaviours.

## Introduction

The prevalence of cannabis users in Australia has increased over recent years (*Australian Institute of Health and Welfare*; 1). Figures from a 2019 national survey indicate that over a third (36%) of Australians aged 14 and over have used cannabis in their lifetime, whilst 11.6% had used in the prior 12 months [1]. Recent research has also suggested that approximately half (51.0%) of Australian cannabis users may drive following cannabis consumption [2], which is of particular concern, given that a recent systematic review has identified a 2.5 times higher likelihood of crashing when driving under the influence of cannabis [3]. In addition to the issue of driving behaviour, cannabis is often considered a gateway drug, in that it has been noted to increase the likelihood of using other illicit substances by up to five times [4]. This raises further concern for the increased: likelihood of poly-substance consumption, inability to judge one’s impairment [5], tendency to drive impaired, and therefore a significantly heightened risk of crashing [6]. Given the highlighted concerns, it is evident that further research is needed to better understand what situational and psychological factors may influence cannabis users to engage in impaired driving behaviours and the consumption of other, potentially more debilitating substances.

The gateway hypothesis suggests that the early onset use of cannabis can lead to an increased likelihood of using other drugs [7, 8]. Whilst some investigators take the perspective that the link between cannabis use with other substance use is merely situational [7], there is another argument that developing a dependence on one drug may lower the threshold for developing dependencies for other substances [9]. In support, research has suggested that heavy and early-onset cannabis use is associated with the development of dependence on various other drugs later in adult life, including opioids [10], methamphetamines [11], and psychoactive substances [8].

According to Schultz, Frohe [12], cannabis is a commonly used but dysfunctional means to cope with negative emotions, particularly among adolescents. This is because using cannabis to cope has been linked with heavy use and a tendency for dependence on the drug [13, 14]. Weiss, Sullivan [15] further explain that individuals who have limited self-regulatory skills and cannot adequately deal with negative emotional experiences, will more likely turn to risky behaviours such as substance use, as an alternative and more immediate means of coping. Recent reviews have highlighted that dysfunctional emotion regulation strategies (e.g., rumination) and self-regulatory deficits are linked with decreased well-being and an increased likelihood for substance use disorder [16, 17]. Further, in a longitudinal-based study following population-level health data from adolescence to adulthood, it was shown that early onset cannabis use typically led to increased consumption patterns of other drugs in later life [8]. However, the authors also found that cannabis users who sought mental health services at a young age were less likely to develop further substance use behaviours [8].

Although research has indicated that those who lack self-regulatory capabilities are more inclined to adopt substance use, additional research also suggests that chronic and heavy substance use can lead to psychological complications, by further reducing the capacity to regulate and express emotions effectively [18, 19]. Two potential causes for these self-regulatory difficulties are that first, chronic and problematic substance use increases the tendency to engage in problematic thinking (e.g., rumination) styles [20] and second, it also reduces neurocognitive performance in the areas of attention, inhibition, memory, and decision making [21]. The prevalence of psychological conditions, such as depression, anxiety, anger, and psychosis, have been noted as high among substance using populations [22–24].

In contrast to the negative effects of cannabis on psychological functioning, it can also be prescribed as a medicine to treat psychological conditions. Although the current growing body of evidence is limited, anecdotal findings have indicated that the various forms of the medication are effective in reducing symptoms associated with a range of psychological conditions (e.g., stress) and disorders (e.g., post-traumatic stress disorder; [25–27]). However, in conjunction with the wider cannabis literature, a question remains regarding the long-term effects of using THC dominated medical cannabis daily, particularly among at-risk individuals prone to psychological dysfunctioning [28]. This issue was highlighted in a recent study, where the majority (58.0%) of the medicinal users reported using both medicinal and black-market cannabis, and that among the medicinal-only participants, over a quarter (28.4%) met the criteria for cannabis use disorder [29]. Alternate research has also identified an overlap between black-market and medicinal users’ psycho-social motives for using cannabis [30–32]. Regardless of the motive however, findings have indicated that using for any psycho-social reason (e.g., enjoyment, enhancement, conformity, coping) may increase the likelihood cannabis use, dependency [33, 34], and mental health issues (30).

Regardless of their motives, populations characterised by substance misuse and emotion dysregulation have demonstrated a tendency towards engagement in risky [35, 36], anti-social [37, 38], and impulsive behaviours [39]. This means that heavier users, particularly poly-substance users, may be at an increased risk of impaired driving. Such patterns were seen in recent qualitative research [31], which identified three groups of substance users, based on their patterns of substance use, attitudes, and relative offending: (a) sporadic recreational users, who used a variety of illicit drugs for social reasons, and drove impaired depending on situational factors, (b) frequent recreational users, who’s drug patterns had evolved to daily cannabis, methamphetamine use, as well as other drugs sporadically (for social reasons), and drove impaired without hesitation, and (c) medicinal users, who primarily used (black-market or medical) cannabis (typically daily), and tended to regulate both their use and driving. Of note, cannabis was present among all groups but was consumed for differing reasons. For example, sporadic recreational users typically used it social reasons; frequent recreational users consumed it to cope; and medicinal users consumed it to treat physical and mental health conditions.

### The present study

In sum of the highlighted literature, it is apparent that the ability to regulate negative emotions plays an important role in the onset and maintenance of psychological problems, as those that lack regulatory skills, may be more likely to turn to maladaptive means of coping, such as substance misuse. Further, research has highlighted factors, such as dependency, cannabis use motives, and psychological dysfunctioning that may predispose individuals to engage in other substance use and impaired driving behaviours. Whilst previous research has identified the links between these factors individually, no current study has explored whether in combination, these factors are associated with the engagement of other substance use and driving behaviours, and among cannabis users specifically. Therefore, this study aimed to explore the relationships between substance use patterns and psychological functioning of cannabis users; and explore the cognitive-behavioural differences of medicinal and black-market cannabis users. From these aims, three research questions were developed: (a) are cannabis users more likely to engage in the use of other substances (i.e., alcohol; methamphetamines; *methyl enedioxy methamphetamine* (MDMA); cocaine; psychedelics; heroin), compared to non-cannabis users?; (b) what are the associations between cannabis use patterns, self-regulatory difficulties (i.e., emotion dysregulation), and psychopathology (i.e., anxiety, depression, and anger), with other substance use and impaired driving behaviours?; and (c) how do substance use patterns, self-regulatory difficulties, and psychopathology differ between medicinal and black-market cannabis flower users?

## Method

### Participants and procedure

Following institutional ethics approval from the University of the Sunshine Coast Human Research Ethics Committee (#S231823), an online survey was shared with a sample of active cannabis users. Participants were engaged via a third-party recruitment service (i.e., Footprints Marketing Research), in which they were sourced through a variety of initiatives (e.g., print media, social media, personal invitations) between the 08/06/2023 and the 21/06/2023. To qualify for the study, participants had to reside in Queensland, own a valid driver’s licence, and had to have used cannabis (flower) in the past month. Participation was also restricted to users of flower (i.e., bud) only, to standardise the use patterns for all participants. Further screening was applied to the recruitment process to ensure a balanced sample of users was obtained. Specifically, age, location, gender, and the purpose of use (i.e., recreational; medicinal) were monitored and controlled throughout the recruitment process. After meeting the eligibility criteria, and providing written consent, participants were presented with the survey, which took approximately 15 minutes to complete. As a means of compensation for their time, participants received tokens after completing the survey, which they could exchange for certain rewards on the Footprints Marketing Research website. The data for this study was collected as part of a larger project investigating the influence of psychological dysfunctioning on a variety of driving behaviours and styles.

Of the total sample recruited (*N* = 200), 53.0% were male and the mean age of participants ranged from 18 to 78 years (M_age_ = 35.0 years). Nearly half (43.5%) of the sample used black-market cannabis, whilst 32.0% used medicinal cannabis, and a further 24.5% reported using both medicinal and black-market cannabis. On average, participants reported that they had used cannabis 10.6 days in the past month. Of those who reported using cannabis in the past month, 49.0% reported that they primarily consumed cannabis via joints or blunts, 29.0% used a water pipe (i.e., “bong”), 9.5% used a hand pipe, 9.5% used a vaporiser, and 3.5% used a hookah. Regarding occupation status, 67.0% reported that they worked full-time, 18.5% worked part-time, 1.0% were self-employed, and 13.5% reported that they did not work, were on disability, or were retired. The majority of participants held an open driver’s licence, whilst 14.0% held a provisional licence, 11.0% were learner drivers, and 5.5% held a commercial licence. On average, participants reported spending 15.6 hours driving on the road each week.

In addition to the primary cannabis user sample, substance use data from non-cannabis users was collected and collated from several recent previous research projects to act as comparative data (*N* = 833). This data was collected using advertising services on Facebook and participants were offered to enter a draw to win one of ten $50 vouchers. The collated data consisted of Australian drivers aged between 18 and 90 years (M_age_ = 51.2 years). Just over half of the sample was male (54.1%) and drove an average of 10.8 hours on the road each week. The majority of the sample held an open driver’s licence (82.3%), whilst 8.9% held a provisional licence, 7.8% held a commercial licence, and 1.0% were learner drivers.

### Measures

#### Demographic information

To better identify the sample’s characteristics, several questions were used to assess participants’ gender, age, licence type (i.e., learners, provisional, open, and commercial), average number of hours spent driving on the road each week, and occupational status.

#### Cannabis use patterns

To measure participants’ specific cannabis use patterns, items from the Cannabis Engagement Assessment [40] were used. The items chosen reflected: (a) the age (in years) when their cannabis use began, (b) the total number of years they had been using cannabis, (c) the number of sessions (separated by blocks of two hours) they typically had on days that they did use cannabis, (d) the quantity of cannabis (in grams) they typically used in a day, (e) the number of days they had consumed cannabis in the past month, (f) the typical method of administration, and (g) whether participants used black-market or medicinal (or both) cannabis. For the measurement of the quantity of cannabis used per day, participants were shown an image of various quantities of cannabis alongside bottle caps to help indicate their usage in grams. Unfortunately, it wasn’t clear if some of the responses to this question were referring to grams or milligrams, and thus the item was excluded from the analyses to avoid any misinterpretations of the data.

#### Cannabis dependence

To measure participants’ likely dependence for cannabis, the *Severity of Dependence Scale* (SDS)–cannabis version [41] was utilised. The SDS is a five-item self-report scale that measures psychological dependence on specific substances. Four of the items are related to the frequency of concerns over usage patterns (e.g., “you worry about your use of cannabis”) and scored from 0 (*never or almost never*) to 3 (*always*), whilst one item is related to the perceived level of difficulty that participants thought they would have stopping their use of the substance, and was scored from 0 (“*not difficult at all*”) to 3 (“*impossible*”). The total score ranges from 0 to 15, with scores of three or more indicating potential dependence on cannabis [42]. The SDS–cannabis version has been shown to have a good internal consistency (α = 0.70; [43]) and is a widely used measure of dependence.

#### General substance use and impaired driving frequency

Several questions were developed to assess participants’ substance use and impaired driving behaviours in the past 12 months. Participants were asked to report how often they: (a) used a variety of substances, including cannabis, alcohol, cocaine, methamphetamine, MDMA, psychedelics, and heroin; (b) drove potentially just and well over the blood alcohol concentration limit for the licence they hold; and (c) drove within four hours of taking each of the drugs. Each question was framed within the timeframe of the previous 12 months and was scored using a five-point frequency scale, where 1 = *never*, 2 = *less than monthly*, 3 = *monthly or more*, 4 = *weekly or more*, 5 = *daily or almost daily*.

#### Cannabis use motivations

To ascertain the primary motivation behind participants’ cannabis use, participants were presented with five potential motivations for using cannabis: (a) for enjoyment (“because it feels good”), (b) for personal enhancement (e.g., “because it makes me a better person; creativity; awareness; perspective”), (c) for social enhancement (“because it makes social situations a lot more fun”), (d) for coping (“because it helps me cope with negative feelings [e.g., sadness; anxiety; anger]”), and (e) for conformity (“because I need to fit in with my friends who use it”). The items were inspired by items and subscales present in the Marijuana Motives Measure [33]. Whilst measures of motivation tend to be scored individually, Maslow’s hierarchy of needs outlines that behaviour is multi-motivated, in that it is simultaneously influenced by more than a single need, and that some motives take precedence over others [44]. This highlights a limitation in the current literature, in that the importance of each motive should be ranked to identify the ***primary*** motivator behind substance use behaviours, within the context of other motives. Therefore, for the current study, participants were asked to rank the motivations (from 1 to 5), based on which items might best represent their motives to use cannabis.

#### Emotion dysregulation

To measure emotion dysregulation, the 18-item version [45] of the *Difficulties in Emotional Regulation Scale* (DERS; [46]) was used. The 18-item DERS identifies six domains of emotion regulation difficulties, including: (a) a lack of awareness of emotions (e.g., “I am [not] attentive to my feelings”), (b) a lack of clarity of emotions (e.g., “I have difficulty making sense out of my feelings”), (c) a lack of goal direction during the experience of negative emotions (e.g., “when I’m upset, I have difficulty focusing on other things”), (d) a lack of impulse control (e.g., “when I’m upset, I have difficulty controlling my behaviours”), (e) a non-acceptance of emotions (e.g., “when I’m upset, I feel ashamed with myself for feeling that way.”), and (f) a lack of access to effective regulation strategies (e.g., “I’m upset, I believe that wallowing in it is all I can do”). Participants were asked to rate each item on how often they experience the proposed difficulties on a five-point scale (1 = *almost never*, 5 = *almost always*). The 18-item DERS is a reliable measure, that has demonstrated a good internal consistency both overall (α = 0.91) and across its subscales (α = 0.77 to 0.90; [45]).

#### Psychopathology–anxiety

Symptoms of anxiety were measured using the *Generalised Anxiety Disorder– 7* (GAD-7) by Spitzer, Kroenke [47]. For this scale, participants are presented with seven items representing the incidence of generalised anxiety disorder [48] symptomology (e.g., “worrying too much about different things”), which participants are asked to rate on a 4-point frequency scale (0 = *not at all*; 3 = *nearly every day*), with higher scores reflecting more frequent symptoms of anxiety. While the GAD-7 is normally referenced to a two-week period, the current study asked participants to consider their symptomology in the past month, in order to standardise the timeframes given between the three psychopathology measures. The longer timeframe (i.e., past month) was chosen over the latter, as the study was not using a clinical based sample. The GAD-7 has displayed excellent internal consistency (α = .92; [47]).

#### Psychopathology–depression

Depressive symptoms were measured using the *Patient Health Questionnaire-9* (PHQ-9; [49]), which was designed to reflect criteria for depressive disorders (48). The PHQ-9 asks participants to rate how often they experienced depressive symptoms (e.g., “feeling tired or having little energy”) using a 4-point scale (0 = *not at all*; 3 = *nearly every day*). The contextual timeframe given to participants was also changed from two weeks to four weeks, for purposes of standardisation. The PHQ-9 has demonstrated good internal consistency (α = .86 to .89; [49]), and is a widely used measure of recent depressive symptomology.

#### Psychopathology–anger

Anger symptomology was measured using the *Dimensions of Anger Reactions—Revised* (DAR-R; [50]) which is an updated version of the original Dimensions of Anger Reactions scale [51]. The DAR-R contains seven self-report items relating to anger experiences (e.g., “when I do get angry, I get really mad”) over the past month. For the current study, participants were also asked to rate each item on a 4-point scale (0 = *not at all*; 3 = *nearly every day*) relating to how often they had experienced such anger, with higher scores indicating an increased frequency of anger symptomology. The DAR-R has demonstrated sound internal consistency (α = .85) and test-retest reliability [50].

### Data analyses

The data for this study were collated and analysed using SPSS (version 29). The variables were compiled using the SPSS compute function and the quality of the variables was assessed using descriptive analyses and manual screening of the data. The aggregate scales (except the SDS) were averaged (rather than summed) as mean scores were decidedly more representative of the original scales’ scoring, and therefore, more interpretable, and comparable. The datasets generated during the current study are not publicly available due ethical restrictions but may be available from the corresponding author on reasonable request, subject to ethical approval.

To attend the first research question, frequencies and cross-tabulations were used to explore the proportions and prevalence of substance use behaviours among cannabis and non-cannabis users. The comparative likelihood of using (non-user percentage divided into cannabis user percentage) was calculated to demonstrate the comparative degree of other substance use engagement between the samples. Independent t-tests were also used to compare the frequency of other substance use behaviours between the two samples.

Bivariate correlations were then used to investigate the specific lineal relationships between cannabis use patterns, cannabis use motivations, self-regulatory difficulties, and psychopathology, with substance use and impaired driving behaviours. To understand the multivariate impact and degree to which the variables predicted substance use and impaired driving behaviours, four multiple regressions were performed with the combined substance use and impaired driving behaviours as the dependent variables, and the cannabis use patterns and psychological dysfunctioning variables as independent groups of predictors. Finally, an ANOVA was used to compare the substance use behaviours, cannabis use motivations, and psychological dysfunctioning, between medicinal users, black-market users, and users of both types. Effect sizes for the t-tests (Cohen’s *d*; .20 = small, .50 = medium, .80 = large), correlations (Pearson’s *r*; .10 = small, .30 = medium; .50 = large), regressions (η^2^; .02 = small, .13 = medium, and .26 = large), and ANOVAs (η^2^; .01 = small, .06 = medium, and .14 = large) were interpreted as per recommendations by Cohen [52].

## Results

Prior to the formal analyses, descriptive statistics indicated that the data was adequate, although, there were some deviations from recommended normality values (skew < 2.0, kurtosis < 7.0; [53]) for heroin use frequency (skew = 2.19) and heroin driving frequency (skew = 2.07). However, these were only minor deviations and were considered expectant for the measured behaviours. Therefore, these variables were left to be analysed in their natural form.

### A comparison of substance use between cannabis and non-cannabis users

To compare the differences in substance use behaviours between non-cannabis users and cannabis users, independent t-tests were performed and showed that there were significant differences between the groups for all behaviours including alcohol use frequency (*t* (340) = 32.99, *p* < .001, *d* = .30), methamphetamine use frequency (*t* (201) = 9.07, *p* < .001, *d* = 1.39), MDMA use frequency (*t* (199) = 8.96, *p* < .001, *d* = 1.43), cocaine use frequency (*t* (202) = 8.34, *p* < .001, *d* = 1.27), psychedelics use frequency (*t* (200) = 7.57, *p* < .001, *d* = 1.04, and heroin use frequency (*t* (199) = 6.39, *p* < .001, *d* = 1.03). In addition to the t-tests, frequencies and the comparative likelihood values were generated, also revealing that cannabis users were much more likely to use other substances. Specifically, cannabis users were at least 25 times more likely to have used other drugs daily or almost daily, at least 95 times more likely to use other drugs weekly or more, at least 100 times more likely to use other drugs monthly or more, and at least 14.3 times more likely to use once to a few times per year. The data also indicated that whilst cannabis users were approximately twice as likely to drink alcohol up to a frequency of weekly or more, they were 1.35 times less likely to drink daily or almost daily. Of note, approximately one-fifth of cannabis users were daily users and one in ten users drove within four hours of consuming, daily. The means, standard deviations and specific proportional data are displayed in Table 1.

**Table 1:** The proportions of substance use and impaired driving behaviours among cannabis users.

Substance Type	M	SD	*t*	*p*	*d*	Never	Less than monthly	Monthly or more	Weekly or more	Daily or almost daily
**Cannabis use**	3.45	1.05	-	-	-	0.0%	23.0%	28.5%	29.0%	19.5%
**Cannabis driving**	2.43	1.39	-	-	-	36.0%	22.5%	14.5%	16.5%	10.5%
**Alcohol use–NCU**	2.86	1.38				20.5%	27.1%	12.8%	24.6%	14.9%
**Alcohol use–CU**	3.27	1.19	32.99	< .001	.30	12.0%	14.0%	26.0%	46.0%	11.0%
**Comparative Likelihood**						-1.71	-1.94	+2.03	+1.87	-1.35
**Methamphetamine use–NCU**	1.01	.19				99.4%	0.2%	0.1%	0.1%	.10%
**Methamphetamine use–CU**	1.80	1.22	9.07	< .001	1.39	65.0%	7.5%	14.0%	9.5%	4.0%
**Comparative Likelihood**						-1.53	+37.5	+140.0	+95.0	+40.0
**MDMA use–NCU**	1.00	.06				99.6%	0.4%	0.0%	0.0%	0.0%
**MDMA use–CU**	1.73	1.15	8.96	< .001	1.43	63.5%	16.0%	7.5%	10.0%	3.0%
**Comparative Likelihood**						-1.57	+40.0	+ ∞	+ ∞	+ ∞
**Cocaine use–NCU**	1.02	.19				98.7%	1.1%	0.1%	0.0%	0.1%
**Cocaine use–CU**	1.63	1.03	8.34	< .001	1.27	65.5%	16.5%	10.0%	5.5%	2.5%
**Comparative Likelihood**						-1.51	+15.0	+100.0	+ ∞	+25.0
**Psychedelics use–NCU**	1.01	.08				99.3%	0.7%	0.0%	0.0%	0.0%
**Psychedelics use–CU**	1.61	1.12	7.57	< .001	1.04	72.0%	10.0%	6.5%	8.5%	3.0%
**Comparative Likelihood**						-1.38	+14.3	+ ∞	+ ∞	+ ∞
**Heroin use–NCU**	1.00	.00				100.0%	0.0%	0.0%	0.0%	0.0%
**Heroin use–CU**	1.50	1.11	6.39	< .001	1.03	79.0%	6.5%	5.0%	4.5%	5.0%
**Comparative Likelihood**						-1.27	+ ∞	+ ∞	+ ∞	+ ∞

NCU = Non-Cannabis User; CU = Cannabis User

### The relationships between cannabis use patterns and psychological dysfunctioning with substance use and driving behaviours

Bivariate correlations were performed to determine the specific lineal relationships present between cannabis use patterns and psychological dysfunctioning variables, with other substance use and driving behaviours. Of the cannabis use patterns, the number of sessions per day (*r* = .20 to .34), level of dependency (*r* = .25 to .54), cannabis use frequency (*r* = .15 to .41), cannabis driving frequency (*r* = .17 to .40), the motivation for personal enhancement (*r* = .20 to .34), and the motivation for social conformity (*r* = .22 to .33), had significant small to large positive associations with the frequency of substance use and driving behaviours. In contrast, the motivation for social enhancement (*r* = -.14 to -.26) and motivation for coping (*r* = -.19, to -.32) had significant small to medium negative relationships with substance use and driving behaviours.

Of note, cannabis and alcohol use behaviours demonstrated a lack of significance to the motivation variables. However, this is likely a consequence of ranked scoring, in that if multiple motivations were positively associated with a behaviour, then the weighting of each relationship would cancel the other out. For the relationships involving psychological dysfunctioning, all the self-regulatory variables (*r* = .19 to .48) and psychopathology variables (*r* = .16 to .51) demonstrated significant small to large positive associations with the dependency, cannabis use, other substance use, and impaired driving behaviours, except for a lack of awareness, which showed inconsistent relationships. The associations between psychological dysfunctioning and alcohol use frequency tended to be lower and trended towards non-significance, compared to the other substance use variables. The specific correlations, along with descriptive and reliability statistics (which ranged from good to excellent; α = .74 to .93). are displayed in Table 2. Alternative correlations not conjunctive to the primary aim of this study are displayed in S1 Table.

**Table 2:** Descriptive statistics, reliability coefficients, and bivariate correlations between the variables.

Variables	Descriptive Stats			Cannabis		Alcohol		Meth		MDMA		Cocaine		Psychedelics		Heroin	
	M	SD	α	Use	Drive	Use	Drive	Use	Drive	Use	Drive	Use	Drive	Use	Drive	Use	Drive
***Cannabis Use Behaviours***																	
**Age of onset**	18.96	6.94	-	-.06	.01	.19**	.12	.04	.05	.10	.11	.07	.10	.09	.09	.16*	.09
**Age**	48.02	18.62	-	.01	-.12	-.16***	-.10	-.10	-.07	-.12	-.09	-.12	-.06	-.10	-.07	-.05	-.07
**Total duration of use**	12.34	11.24	-	.24***	.03	.03	-.13	-.08	-.06	-.16*	-.13	-.15*	-.13	-.14*	-.14*	-.13	-.11
**Quantity of use**	1.98	.99	-	.38***	.39***	.10	.34***	.20**	.23***	.28***	.28***	.25***	.26***	.30***	.29***	.22***	.25***
**Dependency**	3.99	3.18	.83	.20**	.30***	.25***	.48***	.34***	.36***	.45***	.51***	.45***	.53***	.45***	.54***	.48***	.53***
**Cannabis use frequency**	1.47	1.07	-	-	.41***	.08	.10	.20**	.19**	.15*	.11	.16*	.11	.17*	.13	.13	.11
**Cannabis driving frequency**	2.43	1.39	-	.41***	-	.17*	.33***	.41***	.40***	.39***	.34***	.36***	.33***	.40***	.31***	.36***	.27***
**Motivation–Enjoyment**	3.97	1.22	-	.02	-.04	.02	.08	.11	.10	.09	.06	.05	.08	.02	.06	.11	.07
**Motivation–Personal Enhancement**	3.12	1.25	-	.02	.07	-.09	.12	.05	.09	.17*	.14	.13	.19**	.15*	.16*	.16*	.18**
**Motivation–Social Enhancement**	2.35	1.12	-	-.07	-.11	-.09	-.26***	-.15*	-.24***	-.24***	-.22**	-.17*	-.22**	-.15*	-.14*	-.20**	-.19*
**Motivation–Coping**	3.52	1.31	-	.09	-.03	-.09	-.27***	-.25***	-.19**	-.30***	-.25***	-.26***	-.31***	-.25***	-.30***	-.32***	-.28***
**Motivation—Conformity**	2.04	1.21	-	-.07	.10	.25***	.25***	.33***	.26***	.28***	.27***	.25***	.26***	.24***	.22**	.26***	.22**
***Psychological Dysfunctioning***																	
**Lack of Awareness**	2.55	1.00	.77	.04	.04	.05	.06	.07	.08	.08	.06	.00	.03	-.02	.03	-.01	.05
**Lack of Clarity**	2.45	1.00	.76	.24***	.32***	.12	.41***	.33***	.39***	.41***	.45***	.40***	.47***	.40***	.44***	.46***	.48***
**Lack of Goal Direction**	2.76	1.07	.78	.25***	.30***	.13	.41***	.31***	.35***	.29***	.35***	.32***	.34***	.34***	.36***	.33***	.34***
**Lack of Impulse Control**	2.56	1.11	.82	.28***	.28***	.19**	.49***	.36***	.40***	.34***	.39***	.33***	.39***	.34***	.38***	.37***	.41***
**Lack of Acceptance**	2.61	1.00	.76	.30***	.28***	.08	.35***	.28***	.32***	.25***	.26***	.20**	.27***	.26***	.30***	.26***	.30***
**Lack of Strategies**	2.60	1.05	.74	.27***	.28***	.11	.41***	.33***	.34***	.34***	.32***	.30***	.35***	.32***	.36***	.34***	.32***
**Anxiety**	1.24	.80	.91	.21**	.23***	.06	.41***	.27***	.33***	.27***	.32***	.28***	.30***	.26***	.31***	.27***	.31***
**Depression**	1.21	.79	.92	.24***	.21**	.10	.37***	.27**	.32***	.24***	.31***	.23***	.28***	.24***	.31***	.25***	.28***
**Anger**	.94	.84	.93	.14*	.29***	.16*	.51***	.37***	.42***	.34***	.41***	.34***	.38***	.34***	.39***	.36***	.39***

NOTE

*p < .05

**p < .01

***p < .001

### The impact of cannabis use patterns and psychological dysfunctioning on substance use and driving behaviours

Four multiple regressions (Table 3) were then implemented to assess the impact that the *cannabis use patterns* and *psychological dysfunctioning* had towards the different substance use and driving behaviours. The substance use and impaired driving frequency variables were totalled to create two dependent variables representing ‘other substance use behaviour’ (α = .88) and ‘impaired driving behaviour’ (α = .92). Durban-Watson values and collinearity statistics were all in acceptable ranges. Only variables which demonstrated to have consistent and meaningful relationships in the bivariate correlations were included in the models.

**Table 3:** Four multiple regressions with cannabis use patterns, and psychological functioning predicting other drug use and driving behaviours.

Predictor	Other Substance Use Behaviour								Impaired Driving Behaviour							
	*B*	*SE*	β	*t*	*R*	*p*	*R* ^*2*^	*F*	*B*	*SE*	β	*t*	*r*	*p*	*R* ^*2*^	*F*
***Cannabis Use Patterns***						< .001	.390	17.53						< .001	.434	21.01
**Cannabis use frequency**	.58	.31	.11	1.85	.13	.066			.64	.44	.09	1.47	.11	.143		
**Quantity of use**	.26	.36	.05	.71	.05	.479			.95	.51	.12	1.87	.13	.064		
**Dependency**	**.66**	**.11**	**.39**	**6.03**	**.40**	**< .001**			**1.02**	**.15**	**.42**	**6.63**	**.43**	**< .001**		
**Motivation–Personal Enhancement**	.25	.32	.06	.78	.06	.435			.47	.45	.08	1.05	.08	.293		
**Motivation–Social Enhancement**	-.56	.36	-.12	-1.55	-.11	.123			-.94	.50	-.14	-1.86	-.13	.065		
**Motivation–Coping**	**-1.05**	**.28**	**-.26**	**-3.70**	**-.26**	**< .001**			**-1.42**	**.40**	**-.24**	**-3.58**	**-.25**	**< .001**		
**Motivation—Conformity**	.61	.36	.14	1.71	.12	.088			.66	.50	.10	1.33	.10	.184		
***Psychological Dysfunctioning***						< .001	.260	8.40						< .001	.328	11.67
**Lack of Clarity**	**1.60**	**.53**	**.30**	**3.05**	**.22**	**.003**			**2.23**	**.72**	**.29**	**3.08**	**.22**	**.002**		
**Lack of Goal Direction**	.62	.71	.12	.88	.06	.380			.68	.98	.09	.69	.05	.488		
**Lack of Impulse Control**	1.18	.75	.24	1.58	.11	.115			1.75	1.03	.25	1.71	.12	.089		
**Lack of Acceptance**	**-1.85**	**.72**	**-.35**	**-2.59**	**-.18**	**.010**			-1.85	.99	-.24	-1.88	-.13	.062		
**Lack of Strategies**	.45	.66	.09	.68	.05	.499			-.09	.91	-.01	-.10	-.01	.918		
**Anxiety**	.03	.86	.00	.03	.00	.976			.63	1.18	.07	.53	.04	.594		
**Depression**	-1.10	.90	-.16	-1.22	-.09	.223			-1.41	1.24	-.14	-1.14	-.08	.256		
**Anger**	**1.45**	**.72**	**.23**	**2.01**	**.14**	**.046**			**2.63**	**.99**	**.28**	**2.66**	**.19**	**.009**		

For the first regression with *cannabis use patterns* predicting other substance use behaviours, the model was significant (*F* (7, 199) = 17.53, *p* < .001) and predicted 39.0% of the variance. Individually, only dependence (β = .39, *t* = 6.03, *p* < .001) and coping motivation (β = -.26, *t* = -3.70, *p* < .001) were significant predictors. In contrast, the second regression with *psychological dysfunctioning* predicting other substance use behaviours was also significant (*F* (8, 199) = 8.40, *p* < .001) and predicted 26.0% of the variance. Individually, a lack of clarity (β = .30, *t* = 3.05, *p* = .003), a lack of acceptance (β = .30, *t* = 3.05, *p* = .003), and anger (β = .30, *t* = 3.05, *p* = .003) were significantly predictive of other substance use behaviours. Overall, the models predicting other substance use behaviours had large effects (Cohen’s *f*^*2*^ = .64; 35, respectively), and the individual effects were small to large in size (*r*^2^ = .02 to .16).

For the third regression with *cannabis use patterns* predicting impaired driving behaviours, the model was significant (*F* (7, 199) = 21.01, *p* < .001) and predicted 43.4% of the variance. Similar to the first regression, only dependence (β = .42, *t* = 6.63, *p* < .001) and coping motivation (β = -.24, *t* = -3.58, *p* < .001) were significant individual predictors. Finally, the fourth regression with *psychological dysfunctioning* predicting impaired driving behaviours was also significant (*F* (8, 199) = 11.67, *p* < .001) and predicted 32.8% of the variance. Individually, only a lack of clarity (β = .29, *t* = 3.08, *p* = .002) and anger (β = .28, *t* = 2.66, *p* = .009) were significant predictors. Overall, the models predicting impaired driving behaviours also had large effects (Cohen’s *f*^*2*^ = .77; 49, respectively), and the individual effects were small to large (*r*^2^ = .04 to .18).

### Comparisons of medicinal and black-market cannabis users

To compare the usage patterns, driving behaviours, and psychological dysfunctioning of medicinal and black-market users, an ANOVA (Table 4) was implemented, and showed that there were significant differences for user’s current age (*Browns-Forsythe* (*BF*; 2, 199) = 7.62, *p* < .001, *η*^*2*^ *=* .011), the total duration of cannabis use (*BF* (2, 199) = 9.37, *p* < .001, *η*^*2*^ *=* .019), the level of dependency (*F* (2, 199) = 3.45, *p* = .034, *η*^*2*^ *=* .034), the motivation for social enhancement (*BF* (2, 199) = 4.10, *p* = .018, *η*^*2*^ *=* .023), drink driving frequency (just over; *BF* (2, 199) = 4.56, *p* = .012, *η*^*2*^ *=* .047), cocaine use frequency (*BF* (2, 199) = 3.47, *p* = .034, *η*^*2*^ *=* .038), cocaine driving frequency (*BF* (2, 199) = 3.68, *p* = .028, *η*^*2*^ *=* .041), heroin use frequency (*BF* (2, 199) = 3.06, *p* = .022, *η*^*2*^ *=* .045), and heroin driving frequency (*BF* (2, 199) = 3.48, *p* = .033, *η*^*2*^ *=* .038). Post-hoc Bonferroni tests were performed to identify the specific intergroup differences, and showed that there were only significant differences between black-market users and users of both cannabis types for: dependency (ΔM = 1.38, *p* = .045), social motivation (ΔM = .53, *p* = .022), drink driving (ΔM = .65, *p* = .007), cocaine use (ΔM = .48, *p* = .025), cocaine driving (ΔM = .48, *p* = .033), heroin use (ΔM = .59, *p* = .008), and heroin driving (ΔM = .51, *p* = .034). Alternatively, the analyses showed that medicinal users were significantly younger than black-market users (ΔM = -6.78, *p* < .001), had a shorter total duration of use than both black-market users (ΔM = -7.31, *p* < .001) and users of both cannabis types (ΔM = 5.63, *p* = .020).

**Table 4:** Means, standard deviations and comparative statistics across cannabis user types.

Variables	Medicinal (*n* = 64)		Black-Market (*n* = 87)		Both (*n* = 49)		ANOVA		
	M	SD	M	SD	M	SD	*F*	*η* ^*2*^	*p*
***Cannabis Use Behaviours***									
**Age of onset**	19.75	6.48	18.40	6.96	18.94	7.51	.69	.007	.501
**Current age**	**31.16**	**8.48**	**37.93**	**13.31**	34.80	9.87	**7.62** ^***BF***^	**.011**	**< .001**
**Total duration of use**	**7.78**	**8.71**	**15.09**	**12.42**	**13.41**	**10.26**	**9.37** ^***BF***^	**.019**	**< .001**
**Quantity of use**	2.00	.98	1.93	1.03	2.04	.96	.21	.002	.811
**Dependency**	4.30	3.35	**3.35**	**2.75**	**4.73**	**3.49**	**3.45**	**.034**	**.034**
**Cannabis use frequency**	3.47	1.02	3.36	2.75	4.73	3.49	.80	.008	.450
**Cannabis driving frequency**	2.34	1.25	2.36	1.44	2.67	1.48	1.00	.010	.371
**Motivation–Enjoyment**	3.77	1.35	3.99	1.14	4.18	1.17	1.67	.017	1.92
**Motivation–Personal Enhancement**	3.16	1.26	2.93	1.12	3.41	1.27	2.37	.023	.096
**Motivation–Social Enhancement**	2.28	1.05	**2.57**	**1.21**	**2.04**	**.98**	**4.10** ^***BF***^	**.023**	**.018**
**Motivation–Coping**	3.67	1.35	3.61	1.28	3.18	1.27	2.29	.011	.104
**Motivation—Conformity**	2.13	1.20	1.90	1.23	2.18	1.19	1.12	.038	.330
***Other Substance Use Behaviours***									
**Alcohol use frequency**	3.34	1.10	3.15	1.28	3.39	1.15	.83 ^*BF*^	.008	.436
**Drink driving frequency–Just over**	1.91	1.19	**1.60**	**1.08**	**2.24**	**1.32**	**4.56** ^***BF***^	**.047**	**.012**
**Drink driving frequency–Well over**	2.02	1.34	1.57	1.15	2.04	1.46	2.82 ^*BF*^	.030	.063
**Methamphetamine use frequency**	1.70	1.03	1.76	1.29	2.00	1.34	.90 ^*BF*^	.009	.409
**Methamphetamine driving frequency**	1.69	1.14	1.59	1.19	1.84	1.25	3.34	.007	.498
**MDMA use frequency**	1.83	1.25	1.51	.89	2.00	1.34	2.99 ^*BF*^	.033	.054
**MDMA driving frequency**	1.77	1.22	1.38	.89	1.80	1.40	2.68 ^*BF*^	.030	.072
**Cocaine use frequency**	1.72	1.19	**1.41**	**.76**	**1.90**	**1.18**	**3.47** ^***BF***^	**.038**	**.034**
**Cocaine driving frequency**	1.66	1.14	**1.28**	**.80**	**1.76**	**1.27**	**3.68** ^***BF***^	**.041**	**.028**
**Psychedelics use frequency**	1.73	1.17	1.38	.92	1.84	1.30	3.04 ^*BF*^	.033	.051
**Psychedelics driving frequency**	1.63	1.11	1.32	.90	1.76	1.35	2.57 ^*BF*^	.028	.080
**Heroin use frequency**	1.50	1.02	**1.29**	**.86**	**1.88**	**1.47**	**3.06** ^***BF***^	**.045**	**.022**
**Heroin driving frequency**	1.64	1.20	**1.26**	**.88**	**1.78**	**1.36**	**3.48** ^***BF***^	**.038**	**.033**
***Psychological Dysfunctioning***									
**Lack of *Awareness***	2.63	.96	2.49	1.05	2.54	.97	.33	.003	.717
**Lack of Clarity**	2.41	1.03	2.41	.94	2.56	1.06	.44	.004	.645
**Lack of Goal Direction**	2.57	1.13	2.83	1.04	2.87	1.03	1.42	.014	.245
**Lack of Impulse Control**	2.56	1.17	2.55	1.10	2.59	1.08	.02	.000	.976
**Lack of Acceptance**	2.48	1.01	2.66	1.00	2.68	.99	.77	.008	.465
**Lack of Strategies**	2.52	1.06	2.63	1.06	2.65	1.04	28	.003	.755
**Anxiety**	1.13	.76	1.29	.81	1.28	.83	.86	.009	.427
**Depression**	1.09	.76	1.30	.81	1.19	.79	1.26	.013	.286
**Anger**	.88	.81	.93	.87	1.03	.84	.44	.004	.645

NOTE: ^BF^ = Browns Forsythe used

## Discussion

The purpose of this study was to explore the relationships between cannabis use patterns and psychological dysfunctioning, with the use of other substances; and explore the cognitive-behavioural differences of medicinal and black-market cannabis users. In light of these aims, this project intended to answer three research questions: a) are cannabis users more likely to engage in the use of other substances compared to non-cannabis users?; (b) what are the associations between cannabis use patterns, self-regulatory difficulties, psychopathology with other substance use and impaired driving behaviours?; and (c) how do the substance use patterns, self-regulatory difficulties, and psychopathology differ between medicinal and black-market cannabis flower users? To achieve this, a sample of active cannabis users and non-cannabis users, completed an online survey.

### Frequencies and comparative likelihood of other substance use

In regard to the first research question, the results showed that compared to non-cannabis users, cannabis users were far more likely to have used other substances in the past 12 months and have used such substances more frequently. The findings indicated that cannabis users were far more likely to use other substances and do so more frequently. This finding is similar to previous research that indicated a high comparative prevalence between cannabis and the use of other drugs [4]. However, while cannabis users were more likely to consume alcohol sporadically, they were found to be less likely to drink daily or almost daily. This may have been because daily cannabis users may be more dependent on cannabis, or just prefer to consume the drug over alcohol, which is a highly prevalent substance and used commonly among the Australian general population [1]. While these findings are cross-sectional and do not necessarily implicate cannabis as a gateway drug, they do highlight the increased likelihood for cannabis users to engage in the consumption of other substances. As highlighted in prior research, cannabis also tends to be used often and for various reasons (e.g., recreationally; medicinally), even after other substances have been adopted [31].

### Associations between cannabis use and other substance use behaviours

In addition to highlighting the increased tendency for substance use, the findings associated with research question two also identified specific aspects of cannabis use that led to a higher frequency of using and driving on other substances. In particular, quantity of use (sessions per day), and demonstrating a likely dependency were the most prominent and impactful use patterns associated with other substance use and driving behaviours. This is consistent with previous research [13, 14], and has been associated with a tendency to use cannabis as a means to cope with negative emotions [12]. As a result, such individuals are more likely to engage in other substances to cope or to improve psychological functioning in specific situations eliciting negative emotions (e.g., social interactions [9]). In addition to substance use alone, a characteristic of substance dependency is that users will continue to use, regardless of the consequences associated with their use [48]. As such, dependent users may be more likely to drive following substance use because there is a lack of separation between the use of the substance and daily activities broadly, of which driving is just one of many. However, this may also be because dependent users develop a tolerance to their typical substances, and therefore hold lower perceptions of risk [31, 54], and have greater difficulty detecting their impairment [5].

The frequency of cannabis use was not a strong nor reliable predictor of other substance use behaviours. However, as previously identified, some users who consume cannabis for medicinal reasons may regulate their use and driving behaviour [31]. For example, some cannabis users may use daily, but as a sleep aid, and thus the impact of cannabis would likely be having a limited effect on their daily functioning. This also potentially highlights that using cannabis strictly for reasons outside of psycho-social motives, such as for physical ailments, may have a different relation to the use of other substances. If users are consuming for the specific medicinal properties of cannabis, they are not necessarily using as coping or enhancement mechanisms, which other substances can also provide.

Whilst literature currently identifies the degree of psycho-social and physical motives among various cannabis users [30, 55], research on the comparison of such motives and in relation to psychological issues and problematic substance use, is sparse. In contrast to previous research which has indicated motivation may lead to more frequent substance use behaviours [30, 32], the current study showed that after accounting for the hierarchy of motives, other substance use behaviours (excluding cannabis and alcohol) were negatively related to the motivation for coping and social enhancement, positively related to the motivation for personal enhancement and conformity, and not related to the motivation for enjoyment. However, the current study implemented a ranked motivation scale, meaning that if participants typically scored high in other substance use behaviours, and also chose a motivation as more relevant than another, the secondary motivation would be inclined to represent a negative relationship. Further, if a motivation was often chosen as both a primary and secondary motivator, the relationships would trend towards neutrality.

In light of the unique scoring dynamics, the results support the importance of social factors (i.e., peer pressure) that is contributing to other substance use and impaired driving behaviours, outside of cannabis and alcohol. This is consistent with themes in previous research which highlight the use of recreational ‘party’ drugs (e.g., cocaine; MDMA) as being more prominently used in social settings [31]. Although the results suggest that those who primarily use cannabis to cope are less likely to use such recreational drugs and drive, this does not necessarily apply to cannabis and alcohol, which are commonly consumed as part of everyday life, and daily [1]. Of interest, coping motivations were negatively related to conformity motivations in the current study. As per previous research, coping is characteristic of older substance users [56], whilst peer pressure is signature of younger populations [57]. Such patterns indicate that age may be playing a moderating role in the relationship between motivation and substance use.

### Associations between psychological dysfunction and other substance use behaviours

The findings also demonstrated that emotion regulation difficulties and psychopathology (i.e., anxiety, depression, and anger) were positively associated with likely cannabis dependency, the frequency of substance use and impaired driving behaviours. These associations are supportive of past research which highlights the links between psychological dysfunctioning with substance misuse [16, 17, 19] and risky driving behaviours [35, 58]. At the univariate level, psychopathology (i.e., anxiety, depression, and anger) and a lack of: goal direction, impulse control, access to effective strategies, acceptance of emotions, and understanding of emotions, were all positively related to other substance use. Of interest, a lack of emotional awareness was the only self-regulatory variable not significantly related to substance use behaviours. However, this may be because having a low awareness of emotions does not necessarily result in dysfunction, as having excessive monitoring and cognitive self-consciousness can also lead to a loss of cognitive control and the development of psychopathology [59].

At the multivariate level, only a lack of emotional clarity and anger remained significant predictors of substance use and impaired driving behaviours. From a statistical perspective, this significance may be the resulting uniqueness of the variables compared to their counterparts. However, from a theoretical translation, the results could imply that substance users tend to lack understanding of their emotions, as this is also a primary feature of anger [60]. Low emotion intelligence (which is derived from understanding one’s emotions) and anger have been consistently been linked to substance use disorders in the literature [17, 61, 62], largely because individuals lacking emotional intelligence are primed for impulsive decision making and have limited means to regulate their emotions.

### Comparisons of black-market and medicinal cannabis users

When approaching the third research question, it was shown that apart from age and the total duration of use, medicinal users did not differ from recreational users in terms of substance use behaviours, impaired driving behaviours, psycho-social motivations for using cannabis, and psychological dysfunctioning. However, recreational users did report significantly less engagement in alcohol, cocaine, or heroin use; and reportedly drove significantly less than those who used both recreational and medicinal cannabis (who thus appeared to be the most problematic group). These findings are partly supportive of previous research, which highlight that medicinal users are significantly more likely than recreational users to engage in substance use behaviours, problematic cannabis use, and exhibit psychopathology [55], Further, in conjunction with themes identified by Love, Rowland [31] and consistent across cannabis user type motives [30, 32], substance use and driving patterns do not appear dependent on whether participants used medicinal or recreational cannabis, but as to the reason they used the drug in the first place. This theme in the research suggests that there may be a substantial proportion of medicinal users who are prone to psychological vulnerability, have a dependency for cannabis (among other substances), and are being prescribed for daily cannabis use. Such implications should be considered in legislations surrounding the regulation and screening of participants applying for prescribed medicinal cannabis.

### Implications, limitations, and future directions

Overall, this study has highlighted that cannabis users are at a significantly higher likelihood to use other substances and has identified potential underlying factors that might influence cannabis users’ tendency for other substance use and impaired driving behaviours. In particular, the inability to regulate emotions, combined with heightened experiences of anxiety, depression and anger were associated with greater propensity for likely dependency. These factors were also linked with a greater likelihood to engage in the use of other substances and impaired driving behaviours. Using cannabis primarily for social conformity was also identified as a prominent motive leading to greater substance use and impaired driving behaviours. This finding was attributed to the increased situational risk These findings have important implications for understanding likely drug dependency, which is tied to impaired driving behaviours. Indeed, the findings identified the significance of psychological dysfunctioning as antecedents to these behaviours, which is important as the role of emotional dysregulation has seldom been a focus when examining impaired driving behaviour. Further, the findings also exhibit practical implications, as they suggest that current interventional approaches (e.g., educational programs) may benefit from the additional implementation of self-regulatory concepts to help extend the duration of interventional effects on behaviour.

In terms of the limitations, the measures were self-report, which may introduce some level of bias especially concerning questions relating to motivations, substance use, and impaired driving behaviours. It is also important to note that the item measuring the quantity of cannabis use (in grams) returned questionable data and could not be used in the analyses. Similarly, the quantity (only frequency) of use for other substances was not recorded. Further investigations into the degree to which users engage in their respective substances may provide further insights into the influences of psychological dysfunction on dependence. Third, the current study was focused on the psycho-social motives of users and did not consider motives of using for physical ailments (e.g., pain) or medical reasons (e.g., Parkinson’s disease). This limited the implications regarding the differences in motives between medicinal and black-market users and should be considered in future research. A final limitation of the study was that the design was cross-sectional, which is considerable when exploring the tendency for cannabis to act as a transitional drug. However, this study was exploratory and thereby aimed to capture a snapshot of current cannabis users and their current use patterns, in relation to their psychological functioning.

Given the significance of the findings, future research should consider longitudinal and interventional-based designs to investigate the consumption patterns and psychological functioning of various users, and whether the addition of teaching self-regulatory skills to existing interventions may be beneficial for wider implementation. In the context of cannabis use and driving, two primary forms of intervention could benefit from the implementation of self-regulatory concepts. The first being educational programs to younger generations, specifically, teaching skills on how to effectively regulate emotions, rather than suppress them through more maladaptive means, such as substance use. Alternatively, the implementation of brief self-regulatory training methods could be applied to interventions for existing offenders and substance users. Currently, there are several evidence-based brief interventions (e.g., mindfulness-based stress reduction; attention training technique; metacognitive training modules), which could be implemented to essentially improve self-regulatory skills and subsequent psychological functioning, and therefore reduce the need to depend on substances for coping or social comfort. Regardless of the approach, it is evident that psychological-based interventions could complement current road safety initiatives and reduce the load of finite roadside policing resources.

## Supporting information

S1 Table Secondary correlations not displayed in Table 2. (DOCX)
