# Supplementary material for: Is cannabis a slippery slope? Associations between psychological dysfunctioning, other substance use, and impaired driving, in a sample of active cannabis users
Source: PLoS One. 2024 Oct 9;19(10):e0310958. doi: 10.1371/journal.pone.0310958 (PMC11463771; doi:10.1371/journal.pone.0310958)
Supplement: S1 Table — (DOCX) [file pone.0310958.s001.docx]

**Supplementary Table 1.** *Secondary Correlations not Displayed in Table 2*

| **Variables** | **1** | **2** | **3** | **4** | **5** | **6** | **7** | **8** | **9** | **10** | **11** | **12** | **13** | **14** | **15** | **16** | **17** | **18** | **19** |
| --- | --- | --- | --- | --- | --- | --- | --- | --- | --- | --- | --- | --- | --- | --- | --- | --- | --- | --- | --- |
| 1. Age of onset |  |  |  |  |  |  |  |  |  |  |  |  |  |  |  |  |  |  |  |
| 1. Age | -.14^*^ |  |  |  |  |  |  |  |  |  |  |  |  |  |  |  |  |  |  |
| 1. Total duration of use | .29^***^ | .65^***^ |  |  |  |  |  |  |  |  |  |  |  |  |  |  |  |  |  |
| 1. Quantity of use | -.11 | .01 | -.09 |  |  |  |  |  |  |  |  |  |  |  |  |  |  |  |  |
| 1. Dependency | .04 | -.04 | -.10 | .44^***^ |  |  |  |  |  |  |  |  |  |  |  |  |  |  |  |
| 1. Cannabis use frequency | -.06 | .24^***^ | .01 | .38^***^ | .20^**^ |  |  |  |  |  |  |  |  |  |  |  |  |  |  |
| 1. Motivation – Enjoyment | .01 | .02 | -.02 | .01 | -.08 | .02 |  |  |  |  |  |  |  |  |  |  |  |  |  |
| 1. Motivation – Personal Enhancement | -.07 | -.06 | .04 | .10 | .08 | .02 | .32^***^ |  |  |  |  |  |  |  |  |  |  |  |  |
| 1. Motivation – Social Enhancement | -.04 | -.13 | -.11 | -.20 | -.15 | -.07 | -.11 | -.38^***^ |  |  |  |  |  |  |  |  |  |  |  |
| 1. Motivation – Coping | -.07 | .09 | .19^**^ | .05 | -.07 | .09 | -.49^***^ | -.06 | -.16^***^ |  |  |  |  |  |  |  |  |  |  |
| 1. Motivation - Conformity | .16^*^ | .06 | -.13 | .03 | .21^**^ | -.07 | -.05 | -.30^***^ | -.26^*^ | -.38^***^ |  |  |  |  |  |  |  |  |  |
| 1. Lack of Awareness | -.09 | .02 | .02 | .16^*^ | .18^*^ | .04 | .04 | .01 | -.16^*^ | -.08 | .19^**^ |  |  |  |  |  |  |  |  |
| 1. Lack of Clarity | .06 | -.09 | -.13 | .32^***^ | .43^***^ | .24^***^ | -.04 | .02 | -.14 | -.06 | .21^**^ | .23^**^ |  |  |  |  |  |  |  |
| 1. Lack of Goal Direction | .03 | -.02 | -.15^*^ | .26^***^ | .39^***^ | .25^***^ | .02 | -.13 | -.06 | .06 | .10 | .11 | .69^***^ |  |  |  |  |  |  |
| 1. Lack of Impulse Control | .09 | -.08 | -.17^*^ | .30^***^ | .47^***^ | .28^***^ | -.01 | -.06 | -.13 | .00 | .19^**^ | .20^**^ | .72^***^ | .86^***^ |  |  |  |  |  |
| 1. Lack of Acceptance | .05 | .03 | -.09 | .29^***^ | .30^***^ | .30^***^ | .01 | -.14^*^ | -.06 | .08 | .10 | .11 | .68^***^ | .84^***^ | .84^***^ |  |  |  |  |
| 1. Lack of Strategies | .05 | -.02 | -.12 | .25^***^ | .41^***^ | .27^***^ | .05 | -.07 | -.11 | -.01 | .13 | .14^*^ | .70^***^ | .81^***^ | .83^***^ | .82^***^ |  |  |  |
| 1. Anxiety | -.04 | -.00 | -.18^*^ | .20^**^ | .27^***^ | .21^**^ | -.06 | .04 | -.15^*^ | .09 | .06 | .15^*^ | .61^***^ | .70^***^ | .70^***^ | .67^***^ | .70^***^ |  |  |
| 1. Depression | -.04 | .04 | -.12 | .23^***^ | .32^***^ | .24^***^ | -.01 | .01 | -.13 | .07 | .05 | .23^***^ | .60^***^ | .67^***^ | .71^***^ | .67^***^ | .70^***^ | .85^***^ |  |
| 1. Anger | -.02 | -.06 | -.18^*^ | .32^***^ | .44^***^ | .14^*^ | -.07 | .03 | -.12 | -.04 | .19^**^ | .27^***^ | .67^***^ | .64^***^ | .75^***^ | .66^***^ | .70^***^ | .71^***^ | .76^***^ |

NOTE: ^*^*p* < .05, ^**^*p* < .01, ^***^*p* < .001
